# Retraction: Short-term responses of Spinach (*Spinacia oleracea* L.) to the individual and combinatorial effects of Nitrogen, Phosphorus and Potassium and silicon in the soil contaminated by boron

**DOI:** 10.3389/fpls.2024.1441692

**Published:** 2024-06-12

**Authors:** Frontiers Editorial Office

The journal retracts the 2022 article cited above.

Following publication, concerns were raised regarding the contributions of the authors of the article. Our investigation, conducted in accordance with Frontiers policies, confirmed a serious breach of our authorship policies and of publication ethics; the article is therefore retracted.

This retraction was approved by the Chief Executive Editor of Frontiers. The authors received a communication regarding the retraction and had a chance to respond. This communication has been recorded by the publisher.

